# A single-dose mRNA vaccine encoding the classical swine fever virus E2-ECD induces durable protective immunity in rabbits

**DOI:** 10.1186/s13567-025-01581-1

**Published:** 2025-07-21

**Authors:** Li-Jun Bian, Yu Tang, Fan Yang, Hong Tian, Qin Peng, Ming-Liang Tang, Yi-Zhen Chen, Tian Xia, Shu Li, Hai-Xue Zheng, Hong-Bing Shu, Mi Li

**Affiliations:** 1 Department of Infectious DiseasesMedical Research InstituteFrontier Science Center for Immunology and Metabolism, Medical Research InstituteTaikang Center for Life and Medical Sciences, Zhongnan Hospital of Wuhan University, Wuhan University, Wuhan, China; 2 State Key Laboratory for Animal Disease Control and Prevention, College of Veterinary Medicine, Lanzhou Veterinary Research Institute, Chinese Academy of Agricultural Sciences, Lanzhou University, Lanzhou, China; 3 Gansu Province Research Center for Basic Disciplines of Pathogen Biology, Lanzhou, 730046 China

**Keywords:** Classical swine fever virus (CSFV), mRNA vaccine, E2 protein, protective immunity

## Abstract

Classical swine fever virus (CSFV) spreads in domestic and wild pig populations, causing significant economic losses in the swine industry. Despite the global implementation of live attenuated vaccines, CSFV remains a persistent threat, with sporadic outbreaks reported annually. A major limitation of the current vaccines is safety concerns and the inability to differentiate infected from vaccinated animals (DIVA). The development of DIVA-compliant vaccines is desirable for effectively controlling or eradicating classical swine fever (CSF). Here, we developed two lipid nanoparticle (LNP)-encapsulated mRNA vaccines encoding either the extracellular domain of the CSFV envelope protein E2 (E2-ECD) or its N-terminal 172-amino acid fragment (E2-ECD-N). Immunological assays in mice revealed high antigenicity and long-lasting protective antibody responses from a single dose of either the E2-ECD or E2-ECD-N mRNA vaccine. Notably, both the E2-ECD and E2-ECD-N mRNA vaccines induced robust T cell responses in mice. Furthermore, a single dose (100 μg) of the E2-ECD mRNA vaccine was sufficient to induce long-term (up to 4 months) protective immunity against CSFV infection in rabbits. Our findings highlight the potential of CSFV-E2-based mRNA vaccines as promising strategies for effective CSF prevention and control while enabling DIVA.

## Introduction

Classical swine fever (CSF) is a highly contagious and serious viral disease caused by classical swine fever virus (CSFV) [1]. The disease is prevalent throughout the year and can cause a devastating blow to the pig industry [2, 3]. CSF was first identified in Tennessee, USA, in 1810 and then rapidly spread around the world [4, 5]. Currently, CSF is prevented by systematic prophylactic vaccination and a non-vaccination stamping-out policy. Although large-scale outbreaks have rarely been observed, annual sporadic epizootics or endemics in some regions are continuously being observed [6].

Most of the currently approved vaccines against CSFV are live attenuated vaccines [7], which are hindered by safety concerns, and differentiation between infected and vaccinated animals (DIVA) is impossible on the basis of the antibodies induced [8, 9]. Additionally, most marketed subunit vaccines require high doses and multiple vaccinations [10–12], and these subunit vaccines insufficiently induce cellular immunity [9, 13]. Therefore, the development of a safe and highly efficient vaccine that offers long-term protection with only one dose is urgently needed.

CSFV is a positive-sense, single-stranded and enveloped RNA virus belonging to the genus *Pestivirus* within the family *Flaviviridae*. The CSFV genome consists of only one open reading frame (ORF), which encodes 8 nonstructural proteins (N^pro^, p7, NS2, NS3, NS4A, NS4B, NS5A and NS5B) and 4 structural proteins (Core, E^rns^, E1 and E2) [14, 15]. Among these proteins, the E2 glycoprotein on the virion surface plays a critical role in viral entry. E2 is responsible for binding to host cell receptors and facilitating membrane fusion [16, 17]. As the key player in viral entry and the primary immunogen, E2 contains most of the viral neutralizing epitopes, making it an ideal target for vaccine development [18–20].

mRNA technology is a revolutionary approach in vaccine development that leverages the body's natural protein production process to produce antigens directly [21]. During the COVID-19 pandemic, mRNA technology has emerged as a powerful tool, demonstrating rapid development, efficient production, and excellent safety profiles [22]. These mRNA vaccines exhibit strong immunogenicity and high efficacy against SARS-CoV-2. Recently, mRNA-based vaccines have been developed for different viruses, such as porcine epidemic diarrhea virus, influenza virus, monkeypox virus, Zika virus and rabies virus [23–27].

In this study, we developed two CSFV mRNA vaccines that target either the extracellular domain of E2 (E2-ECD, aa1-337) or the N-terminus of the extracellular domain of E2 (E2-ECD-N, aa1-172). The mRNAs encoding these antigens were encapsulated in lipid nanoparticles (LNPs), and their efficacies were tested in both mouse and rabbit models. Rabbits are sensitive to the CSFV C-strain, which are widely used and recommended by the People’s Republic of China Veterinary Pharmacopoeia as an animal model in place of pigs for testing the efficacy of CSFV vaccines [28, 29]. We demonstrated that both the E2-ECD and E2-ECD-N mRNA-LNP vaccines were highly effective in both mouse and rabbit models. Both E2-ECD and E2-ECD-N mRNA-LNPs induced robust and long-lasting antibody responses in both mice and rabbits. Moreover, a single dose of E2-ECD mRNA-LNP provided long-term (up to 4 months) protective immunity against CSFV infection in rabbits. These findings underscore the potential of mRNA-based vaccines as promising strategies for the prevention and control of CSFV.

## Materials and methods

### Cells and viruses

HEK293T cells were originally provided by Dr Gary Johnson (National Jewish Health). PK-15 cells were obtained from the China Center for Type Culture Collection (CCTCC). Expi293F cells were obtained from the National Collection of Authenticated Cell Cultures (NCACC). HEK293T cells and PK-15 cells were cultured in DMEM (Gibco, #C11875500BT) supplemented with 10% FBS (CellMax, #SA211.02) and 1% penicillin‒streptomycin (Gibco, #15140‒122). Expi293F cells were cultured in suspension in SMM 293-TII expression medium (SinoBiological) without the addition of serum. Expi293F cells were cultured in shaking flasks at 37 °C in an 8% CO₂ incubator at 95 rpm. The CSFV C-strain live attenuated vaccine, utilized as the challenge virus in rabbits, was obtained from the National Center for Veterinary Culture Collection (CVCC). The CSFV Thiverval strain (AV65) for the serum virus neutralization assay was also obtained from the CVCC.

### Antigen sequences and plasmids

The amino acid sequence of the E2 glycoprotein from the CSFV-GZ-2009 strain (GenBank: HQ380231) served as the reference sequence for this study. On the basis of this reference, coding sequences for the extracellular domain of E2 (E2-ECD, aa1-337), the N-terminal fragment of E2-ECD (E2-ECD-N, aa1-172), and the C-terminal fragment of E2-ECD (E2-ECD-C, aa173-337) were codon optimized for efficient mammalian expression and then synthesized by GenScript. The synthesized cDNAs were subsequently cloned and inserted into a pSecTag2 vector modified by our laboratory, which contains an upstream T7 RNA polymerase promoter, a 5’ UTR and a downstream 3’ UTR. To facilitate protein secretion and detection, a DNA fragment encoding a signal peptide was fused in frame to the N-terminus of each antigen coding sequence, and a DNA fragment encoding a flexible linker (GGGGS) and a His-tag was fused in frame to the C-terminus of each antigen coding sequence for protein purification and detection.

### In vitro transcription (IVT)

The transcription templates were amplified by PCR from the plasmids encoding E2-ECD, E2-ECD-N and E2-ECD-C. Poly(d)T-tailed reverse primers were used for PCR to generate transcription templates with poly(A) tails. The linearized DNA templates were transcribed and capped in vitro with a commercial kit (HiScribe T7 mRNA Kit with CleanCap Reagent AG, New England Biolabs, #E2080L) to add a Cap1 structure (N7mGpppAm cap), which is important for mRNA translation and stability. RNase-Free DNase I (Promega, # M6101) was used to remove the residual DNA templates. The resulting mRNA was purified with a Monarch RNA Cleanup Kit (New England Biolab, #T2050L). The integrity of the mRNA was confirmed by agarose gel analysis.

### Transfection and immunoblotting analysis

HEK293T cells were seeded in 12-well plates and transfected with 0.3 μg of mRNA per well using Lipofectamine 2000 transfection reagent according to the manufacturer’s instructions (Invitrogen, #52887). Sixteen hours post-transfection, the cell culture supernatants and cell lysates were analysed by immunoblotting. For reducing SDS‒PAGE, the supernatants or cell lysates were mixed with SDS-loading buffer supplemented with β-mercaptoethanol and boiled at 95 ℃ for 15 min. For nonreducing SDS‒PAGE, the samples were boiled in SDS-loading buffer without β-mercaptoethanol. The antibodies used in this study were as follows: HRP-conjugated 6*His (Proteintech, #HRP-66005), anti-GFP (GeneTex, #GTX113617) and HRP-conjugated goat anti-rabbit IgG (Jackson ImmunoResearch, #111-035-003). Rabbit antisera against CSFV were produced against the CSFV C-strain virus.

### LNP encapsulation and characterization

LNP encapsulation for mRNA was prepared using a microfluidic mixer. Briefly, the prepared mRNA was first diluted in 50 mmol/L sodium acetate buffer (pH 4.0) and then mixed with a lipid mixture in ethanol at a volume ratio of 3:1. The lipid mixture containing cationic ionizable lipid (SM102), 1,2-distearoyl-sn-glycero-3-phosphocholine (DSPC), cholesterol, and DMG-PEG 2000 was dissolved in ethanol. The mRNA-LNPs were subsequently dialyzed against phosphate-buffered saline (PBS) (pH 7.4) and then concentrated using Amicon ultracentrifugal filters (Millipore, USA) with a 100-kD molecular weight cut-off. The concentration and encapsulation efficiency of the mRNAs were measured and calculated with a Quant-it^™^ RiboGreen^™^ RNA Assay Kit (Invitrogen, #R11490). The mRNA-LNP particle size distribution and polydispersity index (PDI) were analysed using a Malvern Nano ZS90.

### Immunizations of mice

For detection of humoral immune responses in mice, female BALB/c mice (6 weeks old) maintained in an SPF facility were randomly divided into nine experimental groups (*n* = 5 mice per group): a control group receiving placebo (PBS), four groups receiving E2-ECD mRNA-LNPs and four groups receiving E2-ECD-N mRNA-LNPs. Mice were immunized with a single intramuscular (i.m.) injection at a dose of 0.5 µg or 5 µg of mRNA-LNP. For the prime-boost regimen, the mice received an initial i.m. injection of E2-ECD mRNA-LNPs (0.5 µg or 5 µg) or E2-ECD-N mRNA-LNPs (0.5 µg or 5 µg), followed by a second injection of the same dose 14 days after primary vaccination. The control group received two PBS injections on Day 0 and Day 14. The mice were intramuscularly vaccinated with mRNA-LNPs in 100 µL of PBS. For all regimens, serum samples were collected at the indicated time points after the initial immunization for antibody analysis.

For detection of cellular immune responses in mice, female BALB/c mice (6 weeks old) were randomly divided into three experimental groups (*n* = 5 mice per group): a control group receiving placebo (PBS), a group receiving a single dose of 5 μg of E2-ECD mRNA-LNPs and a group receiving a single dose of 5 μg of E2-ECD-N mRNA-LNPs. The mice were intramuscularly vaccinated with mRNA-LNPs in 100 µL of PBS. Spleens were collected on day 10 after the initial immunization for evaluation of the cellular immune response.

For detection of long-term humoral immune responses in mice, female BALB/c mice (6 weeks old) were randomly divided into four experimental groups (*n* = 7 mice per group): two groups receiving a single dose of 0.5 μg or 5 μg of E2-ECD mRNA-LNPs and two groups receiving a single dose of 0.5 μg or 5 μg of E2-ECD-N mRNA-LNPs. The mice were intramuscularly vaccinated with mRNA-LNPs in 100 μL of PBS. Serum samples were collected at the indicated time points after immunization for antibody analysis.

### Immunizations and challenge of rabbits

Male New Zealand White rabbits (approximately 2.5 kg) were randomly divided into three experimental groups (*n* = 5 rabbits per group): a control group receiving placebo (PBS), a group receiving a single dose of 100 μg of E2-ECD mRNA-LNPs and a group receiving a single dose of 100 μg of E2-ECD-N mRNA-LNPs. Rabbits were subcutaneously vaccinated with 100 μg of mRNA-LNP in 900 μL of PBS. Serum samples were collected at the indicated times after immunization. At 3 weeks post-immunization, all the rabbits were challenged with 100 rabbit infectious doses (RIDs) of the CSFV C -strain via the marginal auricular vein route, and then, the pyrexia and viral loads were observed. Rectal temperature was monitored and recorded until 4 days post-infection. Fever was considered a rectal temperature ≥ 40 °C that lasted for at least 24 h.

For detection of long-term (up to 4 months) protective immunity against CSFV infection in rabbits, male New Zealand White rabbits (approximately 2.5 kg) were randomly divided into two experimental groups (*n* = 4 rabbits per group): a control group receiving placebo (PBS) and a group receiving a single dose of 100 μg of E2-ECD mRNA-LNP. Rabbits were subcutaneously vaccinated with 100 μg of mRNA-LNP in 900 μL of PBS. Serum samples were collected at the indicated times after immunization. At 18 weeks post-immunization, all rabbits were challenged with 100 RID of the CSFV C-strain via the marginal auricular vein route, and then, the pyrexia and viral loads were observed. Rectal temperature was monitored and recorded until 4 days post-infection. Fever was considered a rectal temperature ≥ 40 °C that lasted for at least 24 h.

### Preparation of recombinant E2-ECD proteins

Briefly, a plasmid expressing E2-ECD was transfected into Expi293F cells. Four days after transfection, the culture supernatant was harvested and dialyzed against PBS. The recombinant E2-ECD was purified with Ni Sepharose high-performance histidine-tagged protein purification resin (Cytiva, #17526801) according to the manufacturer’s protocol. The purified proteins were separated by 10% SDS‒PAGE and stained with a Colloidal Blue Staining Kit (Invitrogen) according to the manufacturer’s protocol.

### Enzyme-linked immunosorbent assay (ELISA)

Anti-E2-specific IgG antibodies were measured by ELISA in mice and rabbits. Briefly, 2 µg/mL of purified E2-ECD was used as a coating antigen on 96-well flat-bottomed microtiter plates (Corning, 655061). After three washes with PBS containing 0.05% Tween 20 (PBST), blocking buffer was added to the plate, which was subsequently incubated for 1 h at room temperature. After another washing step, anti-serum with different dilutions was added and incubated at 37 °C for 1 h. The plates were washed and incubated with HRP-conjugated goat anti-mouse IgG (Jackson ImmunoResearch, 115-035-003) or HRP-conjugated goat anti-rabbit IgG (Jackson ImmunoResearch, 111-035-144) at a 1:5000 dilution for 1 h at room temperature. Tetramethylbenzidine (Invitrogen, N301) was used to develop the ELISA plates, followed by the addition of 2 N sulfuric acid to stop the reactions. The optical density (OD) at 450–570 nm was recorded using a microplate reader (SpectraMax i3x multimode microplate reader, Molecular Devices). The 50% effective concentration (EC_50_) was calculated with GraphPad Prism Version 8.0.2 and fit to a four-parameter logistic curve.

Anti-E^rns^ specific IgG antibodies in rabbits were measured by indirect ELISA. ELISA plates were precoated with E^rns^ antigen derived from a COIBO BIO product (COIBO BIO, CB10746-Pg). Serum samples from the E2-ECD- and E2-ECD-N-immunized rabbits were collected 28 days post-vaccination and tested. Serum samples collected 14 days post-challenge from the CSFV C-strain challenge group served as positive controls. Rabbit serum samples were added at a 1:200 dilution. After incubation at 37 °C for 1 h, the plate was washed and incubated with HRP-conjugated goat anti-rabbit IgG (Jackson ImmunoResearch, 111-035-144) at a 1:2000 dilution for 1 h at room temperature. Tetramethylbenzidine (Invitrogen, N301) was used to develop the ELISA plates, followed by the addition of 2 N sulfuric acid to stop the reactions. The optical density (OD) at 450–570 nm was recorded via a microplate reader (SpectraMax i3x multimode microplate reader, Molecular Devices).

For cytokine detection, mouse splenocytes on day 10 post-vaccination were isolated for IL-2 and IL-4 detection. In brief, 1 mL of the cell suspension (4 × 10^6^ cells/mL) was added to each well of a 12-well plate and stimulated with 30 μg/mL recombinant eukaryotic E2-ECD protein. The cell culture supernatants were collected 72 h after stimulation. The concentrations of IL-2 (BioLegend, 575409) and IL-4 (BioLegend, 431101) in the supernatants were measured with commercially available ELISA kits.

### Virus neutralization assay

Serum samples were heat-inactivated at 56 °C for 30 min, serially diluted (from 5– to 5120–fold) and incubated together with 100 TCID_50_ of the CSFV Thiverval strain (AV65, CVCC) for 1 h at 37 °C. Following the serum-virus incubation, the mixtures (total volume of 200 µL) were added to 4 × 10^5^ PK-15 cells per well in 24-well plates. After 1 h of incubation at 37 °C for virus adsorption, the cells were washed with PBS and then incubated for 7 h in culture medium containing 2% FBS. CSFV RNA was detected and quantified by RT‒qPCR and normalized to *ACTB* levels. The 50% neutralization titre (NT_50_) was calculated with GraphPad Prism Version 8.0.2 and fitted to a nonlinear regression model with a fixed slope based on the viral RNA levels at different serum dilutions.

### Intracellular cytokine staining (ICS) and flow cytometry analysis

Mouse splenocytes were suspended in RPMI-1640 medium containing 10% FBS followed by lysis of red blood cells and stimulation with E2-ECD protein at a final concentration of 100 μg/mL for 48 h. Golgi Stop transport inhibitor cocktail (BD) was added 4 h before cell collection, and the cells were washed with cell staining buffer (PBS supplemented with 1% FBS). The cells were then suspended in Fc Block for 5 min at room temperature prior to surface staining with the following antibodies: anti-CD3-PB (BioLegend, #100214), anti-CD4-FITC (BD, #553046), and anti-CD8-PerCP-Cy5.5 (BD, #553036). After being stained for 30 min at 4 ℃ in the dark, the cells were washed with staining buffer, fixed and permeabilized using a BD Cytofix/Cytoperm fixation/permeabilization kit according to the manufacturer’s instructions. The cells were washed in perm/wash solution, followed by intracellular staining with the following antibodies: IFN-γ-APC (Invitrogen, #17-7311-82) and IL-2-PE (BioLegend, #503808). Finally, the cells were washed in perm/wash solution and then suspended in stain buffer. All samples were analysed with a BD LSRFortessa X-20 cell analyser, and the data were processed using FlowJo software.

### Enzyme-linked immunospot (ELISPOT) assay

IFN-γ (Mabtech, 3321-4AST-2)- and IL-2 (Mabtech, 3441-4HPW-10)-based ELISPOT assays were performed to detect antigen-specific T cell responses. Briefly, spleens were collected from BALB/c mice on day 10 after the initial immunization, and splenocytes were isolated following lysis of red blood cells. Peripheral blood lymphocytes were isolated from rabbits on day 14 and day 120 after the initial immunization following lysis of red blood cells. Mouse splenocytes (5 × 10^5^ cells/well) and rabbit peripheral blood lymphocytes (5 × 10^5^ cells/well) were seeded into the wells of ELLISPOT plates, preincubated with serum-free medium, and stimulated with a final concentration of 30 μg/mL of the E2-ECD recombination proteins or serum-free medium (negative control). After 36 h of stimulation, the cells were removed and washed with PBS five times. The detection antibody was added to the plate, which was subsequently incubated for 2 h at 37 °C. After another washing step, the streptavidin-ALP substrate (for IFN-γ) or the streptavidin-HRP substrate (for IL-2) was added to the plate, and colour development was stopped by washing extensively in deionized water. The spots were counted with a CTL S6 Ultra Analyser.

### qPCR for detection of viral RNA loads

Total RNA was isolated from rabbit spleens and PK-15 cells using RNAiso plus reagent (Takara, 9108Q), reverse-transcribed, and subjected to qPCR analysis to measure the mRNA levels of the tested genes. Lower Ct values were associated with higher viral loads. qPCR was performed using the following primers [30]:

CSFV-100-F: ATGCCCAYAGTAGGACTAGCA

CSFV 192-R: CTACTGACGACTGTCCTGTAC

*ACTB*-F: CTGAACCCCAAAGCCAACCGT

*ACTB*-R: TTCTCCTTGATGTCCCGCACG

### Statistical analysis

For comparisons between two groups, the data were analysed by an unpaired t test. When more than two groups were compared, the data were analysed by one-way or two-way ANOVA. All the statistical analyses were performed with GraphPad Prism 8, and differences with a *P* value < 0.05 were considered statistically significant.

## Results

### Construction and characterization of CSFV-E2-based mRNA vaccines

**Figure 1:**
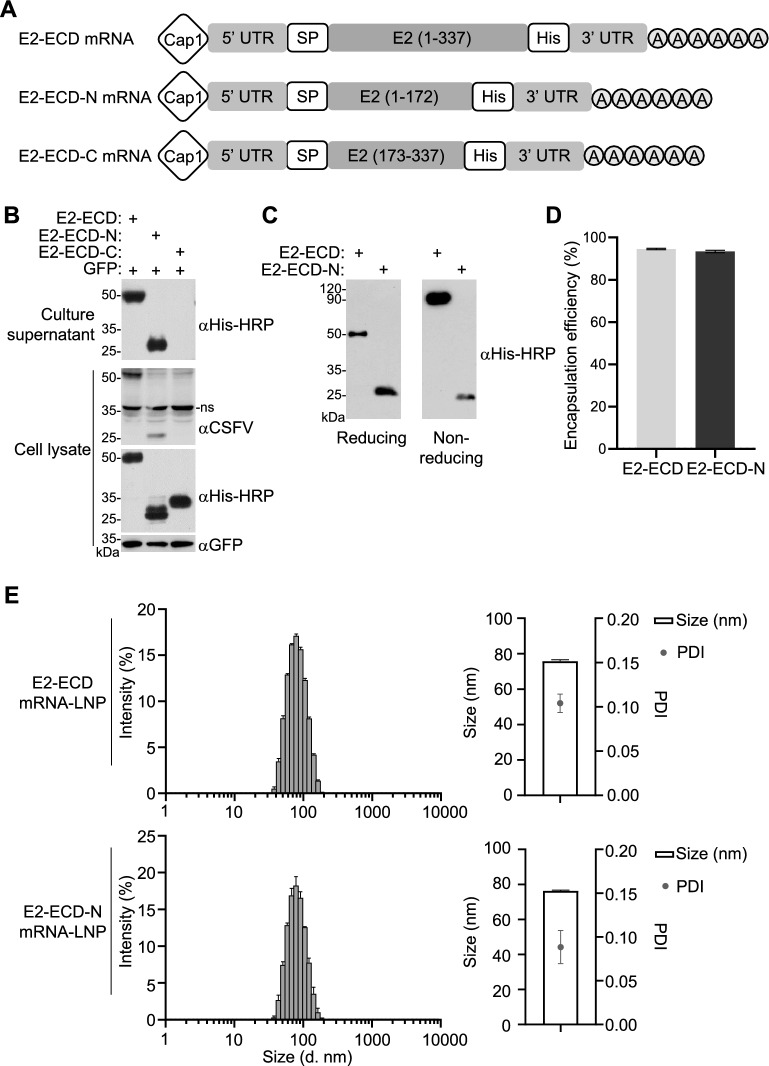
**Construction and characterization of CSFV-E2-based mRNA vaccines.** **A** Illustration of mRNA vaccine constructs, which include the extracellular domain of E2 (E2-ECD, aa1-337), the N-terminus of the extracellular domain of E2 (E2-ECD-N, aa1-172), and the C-terminus of the extracellular domain of E2 (E2-ECD-C, aa173-337). The constructs contain a Cap1 structure at the 5’ end for enhanced mRNA stability and translation, a 5’ untranslated region (5′ UTR), a signal peptide (SP) for secreted protein expression, an antigen coding sequence, a 3’ untranslated region (3′ UTR), and a polyhistidine (His) tag for purification and detection. **B** Antigen expression in HEK293T cells. HEK293T cells were transfected with GFP (0.1 μg), E2-ECD (0.2 μg), E2-ECD-N (0.2 μg), or E2-ECD-C (0.2 μg) mRNAs for 16 h. Protein expression of the indicated mRNAs in the cell lysate and culture supernatant was analysed by immunoblotting analysis with anti-His-HRP and anti-GFP antibodies and rabbit antisera against CSFV. The nonspecific band at ~ 35 kDa is attributed to the cross-reactivity of the primary antibody with a host cell protein. ns, nonspecific. **C** Immunoblotting analysis of the expressed antigens under reducing conditions with β-mercaptoethanol or nonreducing conditions without β-mercaptoethanol. The supernatants of each sample with or without β-mercaptoethanol treatment were loaded onto SDS‒PAGE gels for immunoblotting analysis with anti-His-HRP. **D** Encapsulation efficiency of LNPs. **E** Representative particle size and polymer dispersity index (PDI) of the mRNA-LNPs. The particle size and PDI of the mRNA-LNPs were measured using dynamic light scattering on a Malvern Nano ZS90.

The envelope protein E2 of CSFV is the most potent immunogen for inducing virus-neutralizing antibodies during CSFV infection [31]. Here, we designed three CSFV mRNA vaccines, which express the extracellular domain of E2 (E2-ECD, aa1-337), the N-terminus of the extracellular domain of E2 (E2-ECD-N, aa1-172), and the C-terminus of the extracellular domain of E2 (E2-ECD-C, aa173-337). For all three mRNAs, an mRNA fragment encoding a signal peptide was added in frame to the 5’ end of the antigen coding sequence so that the expressed proteins could be secreted. To efficiently express the antigens, three mRNAs were added with a type 1 (N7mGpppAm) cap and 5’ and 3’ untranslated sequences, and the coding sequences were codon optimized for efficient expression in pigs (Figure 1A).

We examined the expression of the three antigens by transfecting equal amounts of mRNAs into HEK293T cells followed by immunoblotting analysis. The cell lysates and supernatants were analysed using both an anti-His-HRP antibody and rabbit antisera against the CSFV C strain (Figure 1B). In the cell lysates, the expression of E2-ECD, E2-ECD-N and E2-ECD-C was detected by anti-His-HRP, indicating successful translation. However, in rabbit antisera against CSFV, only E2-ECD and E2-ECD-N were detected in the cell lysates. It is possible that this antiserum does not recognize any epitopes in the E2-ECD-C antigen. Secretion of E2-ECD and E2-ECD-N, but not E-ECD-C, into the culture supernatant was observed (Figure 1B). Therefore, E2-ECD and E2-ECD-N were selected for subsequent experiments. The E2-ECDs expressed by mRNAs exist as stable homogeneous dimers under nonreducing conditions, which is similar to the native glycosylated dimeric E2 protein reported in previous studies [32, 33], whereas E2-ECD-N exists as a monomer (Figure 1C). To evaluate whether these mRNAs can be effective vaccines, we encapsulated these mRNAs in lipid nanoparticles (LNPs). The resulting mRNA-LNP particles were found to have an encapsulation efficiency greater than 90% (Figure 1D). The mRNA-LNPs had an average particle size of approximately 75 nm, which is one of the optimal sizes facilitating their internalization and biodistribution [34], and a narrow distribution with a polydispersity index (PDI) below 0.1 indicated that the mRNA-LNP particles were highly uniform (Figure 1E).

### Humoral and cellular immune responses in CSFV-E2-based mRNA-LNP-vaccinated mice

**Figure 2:**
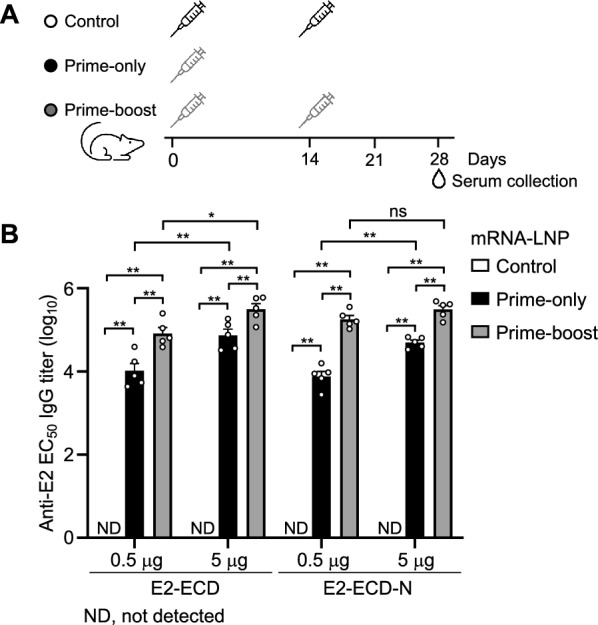
**Humoral immune responses in CSFV-E2 mRNA-LNP-vaccinated mice.** **A** Schematic representation of the experiments. BALB/c mice (*n* = 5 per group) were immunized intramuscularly with 0.5 or 5 μg of E2-ECD or E2-ECD-N mRNA-LNPs via a primer-only or prime-boost regimen. The control group received two doses of placebo (PBS) on Day 0 and Day 14. **B** E2-specific antibody titres in the serum were determined by ELISA on day 28 after immunization. The data are presented as mean ± SEM of five mice per group. The data were analysed using the Mann‒Whitney test with GraphPad Prism 8 (*P < 0.05; **P < 0.01; ND: not detected).

We next determined the immunogenicity of the CSFV E2-ECD and E2-ECD-N mRNA vaccines in a mouse model. The mice were immunized with 0.5 μg or 5 μg of CSFV E2-ECD or E2-ECD-N mRNA-LNP vaccine via intramuscular (i.m.) injection via a prime-only or prime-boost strategy (Figure 2A). E2-specific antibody titres on day 28 after the first immunization were used to evaluate the immunogenicity of the candidate vaccines. The results indicated that inoculation of both E2-ECD and E2-ECD-N mRNA-LNP elicited similarly high levels of antibody titres (Figure 2B). A significant dose response was observed in both the E2-ECD and E2-ECD-N mRNA-LNP prime-only groups, with a marked increase in antibody titres at higher doses. In contrast, the prime-boost groups exhibited a less significant dose‒response relationship. Boost immunization significantly enhanced antibody responses in both the low-dose and the high-dose groups. However, the increase in the antibody response was more significant in the low-dose groups than in the high-dose groups (Figure 2B). These data suggest the potential for interchangeability between dose and boost in influencing antibody responses, particularly in high-dose groups. These results suggest that E2-ECD and E2-ECD-N mRNA-LNPs have comparable immunogenicity and that prime-only inoculation of either elicits high antibody titres.

**Figure 3:**
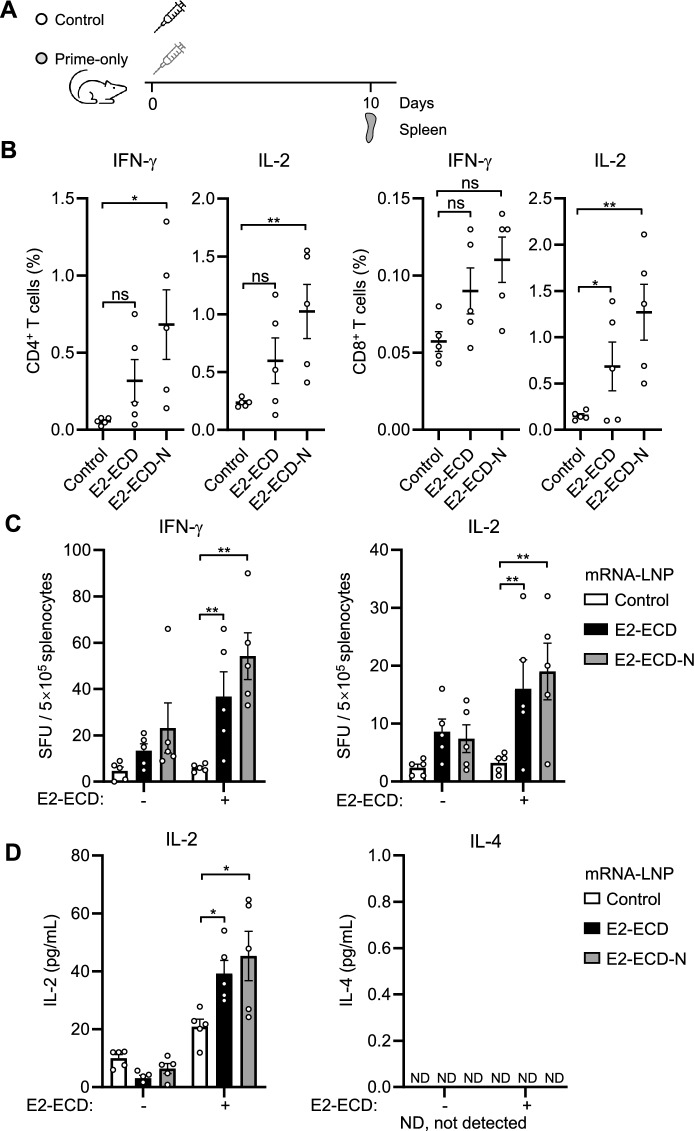
**Cellular immune responses in CSFV-E2 mRNA-LNP-vaccinated mice.** **A** Schematic representation of the experiments. Mice (*n* = 5 per group) were immunized intramuscularly with 5 μg of E2-ECD or E2-ECD-N mRNA-LNPs via a prime-only regimen. The control group received a placebo (PBS). Spleens were harvested 10 days post-vaccination. **B** Frequencies of IFN-γ- and IL-2-producing splenic CD4^+^ and CD8^+^ T cells following E2-ECD protein restimulation were detected via ICS assays and analysed by flow cytometry. Splenocytes were isolated on day 10 and stimulated with the recombinant E2-ECD protein for 48 h. **C** Measurement of CSFV-E2-specific IFN-γ- and IL-2-producing splenocytes by ELISpot assay. Splenocytes (5 × 10^5^) were stimulated with E2-ECD protein for 36 h, and the spots were then counted. **D** Measurement of CSFV-E2-specific IL-2 and IL-4 responses in splenocytes by ELISA. IL-2 and IL-4 secretion by splenocytes after stimulation with the E2-ECD protein for 72 h. The cell culture supernatant was collected for detection by ELISA. The data shown are mean ± SEM of five mice per group. The data were analysed using two-way ANOVA with GraphPad Prism 8 (*P < 0.05; **P < 0.01; ns, not significant; ND, not detected).

CD8^+^ T cell responses induced by attenuated C-strain vaccines play an important role in controlling CSFV infection [35, 36]. The expression of interferon-γ (IFN-γ) and interleukin-2 (IL-2) is a hallmark of T cell activation [37]. To examine antigen-specific T-cell activation, we assessed how the profile of T cell compartment changes using intracellular cytokine staining (ICS) assays. The mice were immunized intramuscularly with 5 μg of E2-ECD or E2-ECD-N mRNA-LNPs via a prime-only regimen (Figure 3A). Spleen cells were collected at 10 days post-vaccination and then stimulated with recombinant CSFV-E2-ECD protein produced in eukaryotic cells. Flow cytometry was used to examine the expression of IFN-γ and IL-2 in E2-ECD-stimulated CD4^+^ and CD8^+^ T cells. The E2-specific CD4^+^ or CD8^+^ T-cell response was weakly induced in the E2-ECD mRNA-LNP group and more significantly induced in the E2-ECD-N mRNA-LNP group (Figure 3B). The numbers of E2-specific IFN-γ- or IL-2-spot-forming T cells were significantly greater in both the E2-ECD and E2-ECD-N groups than in the control group in the ELISpot assays (Figure 3C). T helper type 1 (Th1) lymphocytes secrete IL-2 and IFN-γ and stimulate type 1 immunity. Conversely, Th2 cells secrete IL-4, IL-5, IL-9, IL-10 and IL-13 and stimulate type 2 immunity [38]. To examine the balance of Th1/Th2 immune responses induced by E2-related mRNA vaccines, splenocytes were assayed for cytokines by ELISA. The results revealed a strong IL-2 response but an undetectable IL-4 response in E2-ECD- and E2-ECD-N mRNA-LNP-inoculated mice (Figure 3D). These results suggest that both E2-ECD and E2-ECD-N mRNA-LNPs induce Th1-biased T-cell immune responses in mice.

### E2-ECD and E2-ECD-N mRNA-LNPs elicit long-term humoral immune responses

**Figure 4:**
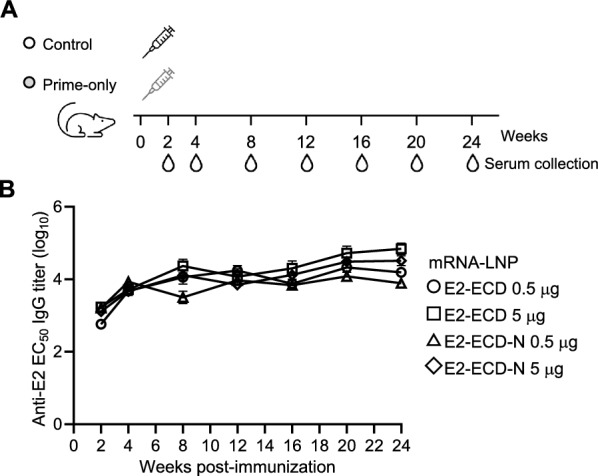
**E2-ECD and E2-ECD-N mRNA-LNPs trigger long-term humoral immune responses.** **A** Schematic representation of the experiments. Mice (*n* = 7 per group) were immunized intramuscularly with 0.5 μg or 5 μg of E2-ECD or E2-ECD-N mRNA-LNPs via a primer-only regimen. Serum samples were collected at the indicated times. **B** E2-specific antibody titres in E2-ECD- and E2-ECD-N mRNA-LNP-vaccinated mice were determined by ELISA. Serum samples were collected at 2- or 4-week intervals for 24 weeks post-immunization, and the E2-specific antibody titres in the serum samples were determined via ELISA. The data shown are the mean ± SEM of seven mice per group.

We next evaluated the duration of the humoral response in E2-ECD- and E2-ECD-N mRNA-LNP-vaccinated mice. The mice were immunized once with two doses of 0.5 μg and 5 μg per mouse. Serum samples were collected at 2- or 4-week intervals for 24 weeks post-immunization to evaluate E2-specific antibody profiles (Figure 4A). Both E2-ECD and E2-ECD-N mRNA-LNP induced robust antibody responses throughout the 24-week period. The high-dose groups presented the highest antibody titres at 24 weeks post-immunization, whereas the antibody titres of the low-dose groups reached the highest levels at 8 weeks post-immunization and remained stable until 24 weeks post-immunization (Figure 4B). These data suggested that a single immunization with either E2-ECD or E2-ECD-N mRNA-LNPs resulted in high antibody titres for at least 6 months.

### E2-ECD mRNA-LNPs provide complete protection from CSFV infection in rabbits

**Figure 5:**
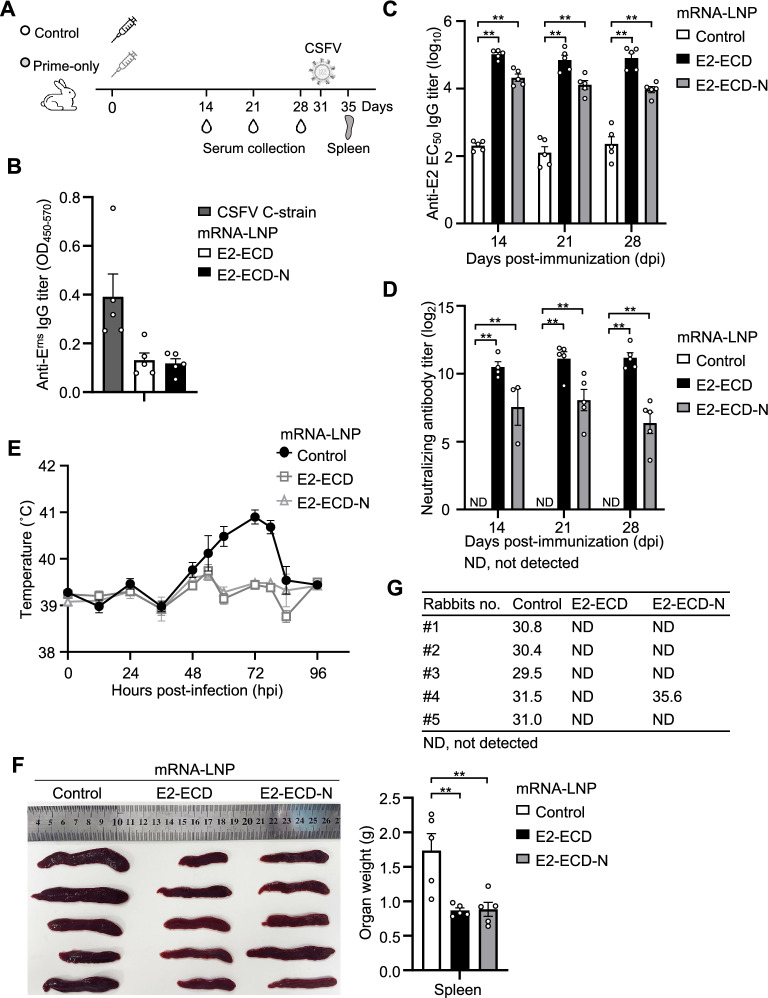
**E2-ECD mRNA-LNPs induce complete protection from CSFV infection in rabbits.** **A** Schematic representation of vaccination, antibody analysis and CSFV challenge in rabbits. Rabbits (*n* = 5 per group) were immunized subcutaneously with 100 μg of E2-ECD or E2-ECD-N mRNA-LNPs via a prime-only regimen. The control group received a placebo (PBS). The serum was harvested on days 14, 21 and 28 post-vaccination. The rabbits were challenged with the CSFV C-strain on day 31 and sacrificed at 4 days post-challenge. **B** The E^rns^-specific antibody titres in rabbit sera were determined by ELISA. Serum samples were collected on day 28 after primary immunization to detect CSFV E^rns^ protein-binding IgG antibody titres. Serum samples from the CSFV-C challenge group, collected 14 days post-challenge, were included as positive controls for infection-induced antibodies. **C** E2-specific antibody titres in rabbit sera were determined by ELISA. Serum samples were collected on days 14, 21 and 28 after primary immunization to evaluate the time course of CSFV E2 protein-binding IgG antibody titre. **D** The neutralizing antibody titres in rabbit sera were determined via a virus neutralization assay. Neutralizing antibodies were measured by their ability to neutralize 100 TCID_50_ of CSFV Thiverval strain (AV65) produced in PK-15 cells. **E** Rectal temperatures of the rabbits following CSFV C-strain challenge were recorded over 4 days. **F** Measurements of the sizes and weights of the spleens following CSFV C-strain challenge. The data shown are mean ± SEM of five rabbits per group. The data were analysed using two-way ANOVA with GraphPad Prism 8 (**P < 0.01; ND, not detected). **G** RT‒qPCR detection of virus loads in spleen samples from the control group, E2-ECD mRNA-LNP group and E2-ECD-N mRNA-LNP group of rabbits after challenge with the CSFV C strain.

We next evaluated the efficacy of E2-ECD and E2-ECD-N mRNA-LNP in rabbits. The rabbits were inoculated with a single dose of 100 μg of E2-ECD or E2-ECD-N mRNA-LNP on day 0. Serum samples were collected at days 14, 21 and 28, and rabbits were challenged with the CSFV C-strain on day 31, as shown in Figure 5A. To assess the DIVA compatibility of the E2-ECD and E2-ECD-N vaccines, we measured E^rns^-specific antibodies in the sera of rabbits on day 28 post-immunization. The results indicated that E^rns^-specific antibodies were easily detected in rabbits infected with the CSFV C-strain for as early as two weeks post-infection but were barely detectable in all the E2-ECD- and E2-ECD-N mRNA-LNP-immunized groups without viral infection (Figure 5B). ELISA revealed that both E2-ECD and E2-ECD-N mRNA-LNP elicited high levels of E2-specific antibodies in rabbits at all time points from days 14–28, and the antibody response was stronger in the E2-ECD group than in the E2-ECD-N group (Figure 5C). Serum samples were further evaluated for their ability to neutralize CSFV. Both the E2-ECD and E2-ECD-N mRNA-LNP groups exhibited robust neutralizing antibody responses, with titres ranging from log_2_ 5 to log_2_ 10 (Figure 5D). Notably, this titre range is comparable to the levels that have been reported to be protective against CSFV challenge in natural hosts [20, 39–41].

Having verified the induction of E2-specific antibodies and CSFV neutralizing antibodies, we performed live viral challenge in the rabbits on day 31 post-immunization. All rabbits were challenged with 100 RIDs of the CSFV C-strain via the marginal auricular vein route, and then, the pyrexia and viral loads were observed. None of the rabbits inoculated with E2-ECD mRNA-LNP developed fever during the observation period of 4 days. One out of five rabbits in the E2-ECD-N group presented with short-term fever for less than 12 h. In contrast, all rabbits in the control group developed fever (≥ 40 °C) for more than 48 h (Figure 5E). Compared with those in the E2-ECD or E2-ECD-N mRNA-LNP group, the spleens in the control group appeared darker and greater in size and greater in weight at 4 days post viral challenge in the rabbits immunized for 31 days (Figure 5F).

To assess the efficacy of the CSFV mRNA vaccine in inhibiting viral replication, RT‒qPCR was performed to detect viral RNA in the spleen. No detectable CSFV RNA was detected in the spleens of rabbits vaccinated with E2-ECD mRNA-LNPs. Only one rabbit in the E2-ECD-N group, which previously presented with short-term fever, presented a low level of viral RNA (Ct value of 35.6). In contrast, all five rabbits in the control group presented high levels of viral RNA (Ct value < 32) (Figure 5G). Taken together, these results demonstrate that a single dose of the E2-ECD mRNA-LNP vaccine provides complete protection against CSFV infection in rabbits.

### E2-ECD mRNA-LNPs induce long-term protective immunity against CSFV in rabbits

**Figure 6:**
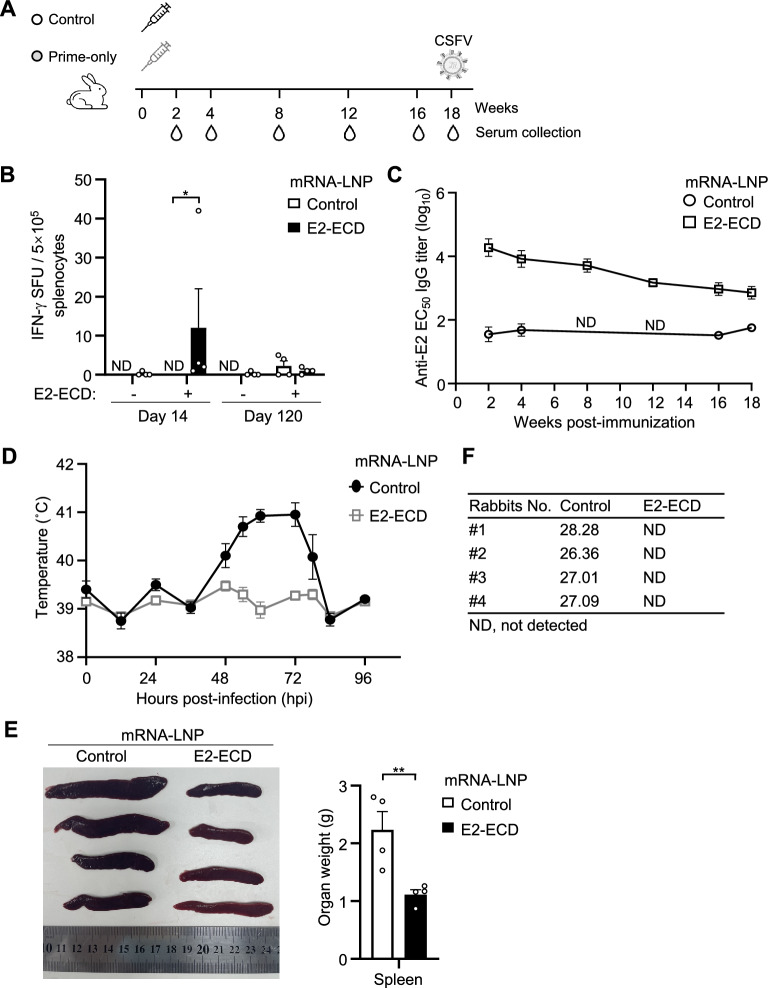
**E2-ECD mRNA-LNPs induce long-term protective immunity against CSFV in rabbits.** **A** Schematic representation of vaccination, antibody analysis and CSFV challenge in rabbits. Rabbits (*n* = 4 per group) were immunized subcutaneously with 100 μg of E2-ECD mRNA-LNP in a prime-only regimen. The control group received a placebo (PBS). Serum samples were collected at the indicated times. The rabbits were challenged with the CSFV C-strain at week 18 and sacrificed at 4 days post-challenge. **B** Measurement of CSFV-E2-specific IFN-γ-producing splenocytes by ELISpot assay. Peripheral blood mononuclear cells (5 × 10^5^) isolated from vaccinated rabbits on days 14 and 120 were stimulated with E2-ECD protein for 36 h, and the spots were then counted. The data were analysed using the Mann‒Whitney test with GraphPad Prism 8 (*P < 0.05; ND, not detected). **C** E2-specific antibody titres in E2-ECD mRNA-LNP-vaccinated rabbits were determined by ELISA. Serum samples were collected at 2- or 4-week intervals for 18 weeks post-immunization, and the E2-specific antibody titres in the serum samples were determined via ELISA. The data shown are the mean ± SEM of four rabbits per group. **D** Rectal temperatures of the rabbits following CSFV C-strain challenge were recorded over 4 days. **E** Measurements of the sizes and weights of the spleens following CSFV C-strain challenge. The data shown are the mean ± SEM of four rabbits per group. The data were analysed using two-way ANOVA with GraphPad Prism 8 (**P < 0.01). **F** RT‒qPCR detection of virus loads in spleen samples from control and E2-ECD mRNA-LNP groups of rabbits after challenge with the CSFV C strain.

We next evaluated the duration of protective immunity against CSFV in E2-ECD mRNA-LNP-vaccinated rabbits. Rabbits were inoculated with a single dose of 100 μg of E2-ECD mRNA-LNP at week 0. Serum samples were collected at 2- or 4-week intervals for 18 weeks post-immunization to evaluate E2-specific antibody profiles, and rabbits were challenged with the CSFV C-strain at week 18, as shown in Figure 6A. To investigate the cellular immunity induced by the mRNA vaccines, peripheral blood lymphocytes were isolated from vaccinated rabbits on day 14 and 120 post-vaccination. The numbers of E2-specific IFN-γ-spot-forming T cells were measured by ELISpot assays. We observed greater numbers of E2-specific IFN-γ spot-forming T cells in the E2-ECD mRNA-LNP group than in the control group on day 14 (Figure 6B). However, the cellular response was barely detectable on Day 120 (Figure 6B). Importantly, we found that E2-ECD mRNA-LNPs induced robust antibody responses 2 weeks post-immunization. The levels of E2 antibodies started to decrease but remained relatively high by 18 weeks post-immunization (Figure 6C).

Having verified the induction of E2-specific antibodies, we proceeded with live viral challenge of the rabbits 18 weeks post-immunization. All rabbits were challenged with 100 RIDs of the CSFV C-strain via the marginal auricular vein route, and then, the pyrexia and viral loads were observed. None of the rabbits inoculated with E2-ECD mRNA-LNP developed fever during the observation period of 4 days. In contrast, all rabbits in the control group developed fever (≥ 40 °C) for more than 48 h (Figure 6D). Compared with those in the E2-ECD mRNA-LNP group, the spleens in the control group appeared darker and greater in size and greater in weight at 4 days post viral challenge in the rabbits immunized for 18 weeks (Figure 6E). We also measured the viral RNA levels in the spleens via RT‒qPCR. The results indicated that CSFV RNA was undetectable in the spleens of rabbits vaccinated with E2-ECD mRNA-LNPs but was easily detected in the control group (Figure 6F). These data suggest that E2-ECD mRNA-LNPs induce durable protective immunity for up to 4 months against CSFV infection in rabbits.

## Discussion

Porcine CSF is a significant global swine disease [42]. It has been well controlled in many intensive swine‐producing countries but often reemerges to cause serious, economically devastating outbreaks [43]. Live attenuated vaccines have been used for decades to control CSF, but they present drawbacks such as safety concerns and the inability to allow for the differentiation of infected and vaccinated animals [44–46]. Other subunit vaccines based on glycoprotein E2 have struggled to induce sufficient cellular immunity [47–49]. In this study, we developed two CSFV mRNA vaccines, E2-ECD and E2-ECD-N mRNA-LNP, one dose of which elicited robust long-lasting humoral immune responses, activated early cellular immune responses and provided sufficient protection from CSFV infection in mouse and rabbit models.

The CSFV E2 protein is a major protective antigen, and the epitopes of the CSFV E2 glycoprotein have been extensively studied [31, 50]. Our data revealed that a single dose of E2-ECD or E2-ECD-N mRNA-LNP elicited high-level antibody titres and had comparable immunogenicity. Notably, these antibody responses were durable in mice, persisting at high levels for at least 6 months following a single immunization.

It has been reported that CSFV-specific IFN-γ production can be detected early after the CSFV C-strain is administered and is correlated with protection against CSFV challenge [51, 52]. These findings suggest that T-cell responses play crucial roles in early defense against CSFV infection. Our research demonstrated that both the E2-ECD and E2-ECD-N mRNA vaccines effectively induced robust E2-specific T-cell responses. CD4^+^ and CD8^+^ T cells are two major lymphocyte subsets involved in adaptive immune responses [53]. Our results revealed that E2-induced expression of IFN-γ and IL-2 was significantly increased in both CD4^+^ and CD8^+^ splenocytes from the spleens of vaccinated animals. Notably, no increase in IL-4 expression was observed, suggesting that these vaccines induce a Th1-based immune response. These findings suggest that E2-based mRNA vaccines have the potential to provide protection against CSFV infection.

A key finding of our study is the protective efficacy demonstrated in the rabbit challenge model. Rabbits are known to be susceptible to the CSFV-C strain. Following challenge with the CSFV C-strain, all control rabbits developed fever and presented evidence of significant viral replication in the spleen and splenomegaly, which is consistent with previous observations in this model [54, 55]. In contrast, a single dose of E2-ECD mRNA-LNP provided complete protection in rabbits. E2-ECD-N mRNA-LNPs also showed high efficacy, protecting most rabbits from fever and significantly reducing viral loads. E2-ECD mRNA-LNPs exhibit greater efficacy than E2-ECD-N mRNA-LNPs do in rabbits, despite their similar immunogenicity in mice, suggesting that E2-ECDs contain key protective epitopes or structural elements that are more critical for protective immunity. These results from the rabbit challenge are highly encouraging, providing strong preclinical evidence that a single dose of E2-ECD mRNA-LNP can induce immunity capable of preventing CSFV infection and replication. These findings align with studies showing that potent immune responses, including neutralizing antibodies and cell-mediated immunity, are essential for protection against CSFV challenge in susceptible models [31, 52].

To further investigate the kinetics and translational relevance of cellular immunity and assess the duration of protective immunity, we evaluated E2-specific IFN-γ spot-forming T cells in vaccinated rabbits on day 14 and day 120 post-vaccination. Consistent with the induction of cellular responses observed in mice, we detected greater numbers of E2-specific IFN-γ spot-forming T cells in the E2-ECD vaccine group than in the control group on day 14 post-vaccination, indicating an early cellular response. However, in line with the expected contraction phase of immune responses, the cellular response became barely detectable on Day 120. Importantly, despite this decline in measurable cellular immunity, the humoral response remained relatively high on Day 120 post-immunization. We proceeded with live viral challenge of these rabbits at this 18-week time point. None of the rabbits inoculated with E2-ECD mRNA-LNPs developed fever during the 4-day observation period post-challenge. In contrast, all the control rabbits developed severe fever for more than 48 h. Furthermore, analysis of the spleens at 4 days post-challenge revealed that CSFV RNA was undetectable in the spleens collected from E2-ECD mRNA-LNP-vaccinated rabbits, whereas it was easily detected in the control group. These results suggest that a single dose of E2-ECD mRNA-LNP induces durable protective immunity against CSFV in rabbits for up to 4 months. These observations suggest that while cellular immunity is rapidly induced and likely plays an important role in early defense, high levels of antibodies appear to play a dominant role in mediating long-term protection against CSFV, which is consistent with previously reported studies on other types of CSFV vaccines [52, 56–58].

While the results in mouse and rabbit models are promising, we acknowledge the inherent limitations of extrapolating these findings directly to natural host pigs. Pigs are the target species and exhibit complex disease pathogenesis upon CSFV infection that is not fully replicated in mice or rabbits. Compared with pigs, mice and rabbits were chosen for this study, primarily as preclinical screening models, owing to their practical advantages, including lower cost, easier handling, reduced biosafety requirements, and faster study timelines. The data obtained from these models are valuable for evaluating basic immunogenicity profiles and providing a proof-of-concept for vaccine-induced immunity in pigs. However, these findings do not constitute a definitive demonstration of efficacy or safety in pigs. A rigorous evaluation of the E2-ECD mRNA-LNP vaccine in a swine challenge model using a virulent CSFV strain is necessary and critical for confirming its protective efficacy, assessing the duration of immunity in the target species, and evaluating its safety under field-like conditions.

Despite the global implementation of live attenuated vaccines, CSFV remains a persistent threat, with sporadic outbreaks reported annually. A major limitation of these vaccines is the inability to differentiate infected from vaccinated animals (DIVA) [59]. To effectively control or eradicate CSF, the marketing of DIVA-compliant vaccines is crucial [60]. It has been reported that the E^rns^-specific antibody response at the early stage of infection is typically quite low [61–63]. In our experiments, we easily detected E^rns^-specific antibodies at two weeks after CSFV infection, suggesting that the sensitivity of our assays is sufficient to detect early or subclinical serological responses. In these assays, we failed to detect E^rns^-specific antibodies in the sera of rabbits on day 28 post-immunization with E2-ECD and E2-ECD-N mRNA-LNPs, suggesting that these vaccines are DIVA compliant. In conclusion, our study suggests that E2-ECD and E2-ECD-N mRNA-LNPs can serve as potential DIVA-compliant vaccines against CSFV and lays a foundation for the development of multivalent mRNA vaccines against CSFV and other serious porcine pathogens. 

## Data Availability

The datasets used and/or analyzed during the current study are available from the corresponding author on reasonable request.
